# The In Vitro Promoting Angiogenesis Roles of Exosomes Derived from the Protoscoleces of *Echinococcus multilocularis*

**DOI:** 10.4014/jmb.2403.03042

**Published:** 2024-05-23

**Authors:** Wenjing Zhou, Xiang Li, Xinqi Yang, Bin Ye

**Affiliations:** Department of Pathogen Biology, College of Basic Medicine, Chongqing Medical University, Chongqing 400016, P.R. China

**Keywords:** *Echinococcus multilocularis*, protoscoleces, exosomes, angiogenesis

## Abstract

Alveolar echinococcosis (AE) is a persistent parasite condition that causes the formation of tumor-like growths. It is a challenge to treat the disease. These growths need neovascularization to get their oxygen and nutrients, and the disease is prolonged and severe. Considerable research has been conducted on exosomes and their interactions with *Echinococcus multilocularis* in the context of immunological evasion by the host. However, the extent of their involvement in angiogenesis needs to be conducted. The primary objective of this investigation was to preliminarily explore the effect of exosomes produced from *E. multilocularis* protoscoleces (PSC-exo) on angiogenesis, to elucidate the mechanism of their roles in the regulation of the downstream pathway of VEGFA activation, and to provide ideas for the development of novel treatments for AE. The study evaluated the impact of PSC-exo increases proliferation, migration, invasion, and tube formation of HUVECs at concentrations of up to 50 μg/ml. In addition, the study sought to validate the findings in vivo. This effect involved increased VEGFA expression at gene and protein levels and AKT/mTOR pathway activation. PSC-exo are crucial in promoting angiogenesis through VEGFA upregulation and AKT/mTOR signaling. This research contributes to our knowledge of neovascularization in AE.

## Introduction

Alveolar echinococcosis (AE) is primarily prevalent in the northern hemisphere and primarily affects humans, who serve as atypical intermediate hosts [1]. *Echinococcus multilocularis* tapeworm larvae cause a serious zoonotic disease that mostly affects the liver, leading to the development of tumor-like lesions [2]. AE presents as an infiltrating tumor with the capacity to metastasize, leading to extensive tissue destruction. If left untreated, the disease carries a significant risk of mortality.

Exosomes are diminutive vesicles, with sizes spanning from approximately 30 to 200 nm, encapsulated within a single lipid bilayer membrane. They vary in size and are distinguished by their high levels of proteins, nucleic acids, lipids, and glycoconjugates [3]. Since 2007, a growing amount of research has highlighted the essential functions that exosomes play in multiple vital biological processes, which include intercellular information transfer [4], reproduction and development [5], metabolism [6], as well as immune response and infection [7, 8] and neovascularization [9]. Exosomes have been increasingly recognized for their significance in research, particularly in parasitic disorders. Many pathogenic parasites, such as *Plasmodium* [10, 11], *Leishmania* [12], *Toxoplasma* [13, 14], and *Schistosoma* [15, 16], produce exosomes during their pathological processes, exerting a pivotal role in the parasite-host interaction and significantly influencing parasite growth, migration, infection, and pathogenesis.

Although extensive research on *E. multilocularis* exosomes and their roles in immune evasion have been conducted, their potential contribution to neovascularization during parasitic infections has been understudied [17, 18]. Currently, the only viable radical treatment for AE is invasive surgical resection [19]. However, as the ability to successfully remove parasitic cysts completely is limited, there is an urgent need to find new therapeutic approaches. In this context, anti-angiogenic therapies targeting vascular endothelial cells (ECs) are receiving increasing attention as a strategy to prevent tumor growth and are considered to be a promising cancer treatment strategy [20]. Therefore, the objective of this study was to preliminarily investigate the function of exosomes produced from *E.multilocularis* protoscoleces (PSC-exo) on human umbilical vein endothelial cells (HUVECs), and the effects on several aspects were then comprehensively evaluated, including cell viability, migration ability, invasion ability, and angiogenesis. Additionally, we sought to elucidate the intricate mechanisms by which PSC-exo achieves this function, to provide novel insights for treating AE. The research provides valuable insights into potential therapeutic targets for the treatment of AE by unraveling the intricate mechanisms through which PSC-exo contributes to this essential biological process.

## Materials and Methods

### Protoscoleces Extraction and Culture

Six months after inoculation with *E. multilocularis* larvae, SD rats were euthanized through dislocated cervical vertebrae. The abdominal cavity was dissected under aseptic conditions, and the intact echinococcus larval tissue was carefully removed. The tissue was then finely shredded and sequentially filtered through 80 and 300-mesh filters to isolate the protoscoleces (PSCs) present in the upper layer of the 300-mesh filter. The PSCs were subjected to multiple rinses with Phosphate Buffer Solution (PBS), followed by isolation and culture for exosomes collection. To ensure the uniqueness of the exosomes source, exosome-depleted fetal bovine serum (Exo-FBS)(VivaCell, China) was used instead of regular serum for culturing to enhance the proliferation of PSCs. The PSCs were then incubated in a cell culture incubator. Every two days, the culture medium was updated while retaining the old version for future use.

### PSCs-Derived Exosomes Isolation and Identification

The supernatant of the PSCs culture was thawed at 4°C one day in advance. It was subjected to centrifugation for 10 min at 4°C and 1,000 rpm. Carefully transfer the resulting supernatant to a new centrifuge tube. The supernatant was centrifuged for 10 min at 300 ×*g*, and after that, it was moved to a clean tube. Once the cellular debris was successfully removed, a third centrifugation step was carried out on the residual liquid at 2,000 ×*g* for 10 min. To ensure the high quality of the PSC-exo, the liquid underwent filtration using a 0.22 μm filter (Millipore, USA) before proceeding with ultracentrifugation. After completing the above procedure, the filtrate underwent centrifugation in a high-speed centrifuge at 140,000 ×*g* for 70 min at 4°C. Following this, a second centrifugation lasting 70 min was conducted to repeat the process. The PSC-exo precipitate was collected and the liquid supernatant was discarded. Then, it was dissolved in PBS. PSC-exós structure and form were investigated using transmission electron microscopy (TEM) (Jeol, Japan). Western blotting revealed the positive markers CD63 and TSG101, and the negative marker Calnexin after their diameter was examined by nanoparticle tracking analysis (NTA).

### Cell Cultivation

HUVECs were bought from Hubei PMTC Cell Technology Co., Ltd. (China). Endothelial cell medium (ECM)(Sciencell, USA) was used to culture HUVECs in accordance with its composition. The culture conditions involved maintaining the cells at 37°C with 5% CO_2_. Upon reaching a confluence of approximately 80% to 90%, the cells were subjected to a 1 to 2-min incubation with 0.25% trypsin (Beyotime, China) for cell passaging.

### RNA Extraction and qRT-PCR Analysis

For the extraction of cellular RNA, Trizol reagent (Beyotime) is employed following the provided instructions for RNA extraction. The process of cDNA synthesis was carried out using ABScript Neo RT Master Mix for qPCR with gDNA Remover (ABclonal, China) after the process of extracting total RNA. For the subsequent qRT-PCR analysis, 2× Universal SYBR Green Fast qPCR Mix (ABclonal) was employed. To ensure precision, each sample was subjected to analysis three times. The data obtained was then scrutinized by comparing the CT values. Tsingke Biological Technology (China) generated the primer sequences, which were as follows: VEGFA forward primer: 5’-TAGAGTACATCTTCAAGCCGTC-3’; VEGFA reverses primer: 5’-CTTTCTTTGGTCTGCATTCACA-3’; GAPDH forward primer: 5’-CGGATTTGGTCGTATTGGG-3’; GAPDH reverse primer: 5’-CGCTCCTGGAAG ATGGTGAT-3’. The mRNA expression fold change was analyzed using accepted techniques as previously reported in research publications [21].

### Extraction of Proteins and Analysis by Western Blotting

The HUVECs were collected, then centrifuged, and rinsed with PBS. The resulting cell precipitate was collected and set aside. To extract the total protein, resuspend the cell pellet in a mixture of freshly prepared RIPA lysis buffer, proteinase inhibitor, and phosphatase inhibitor (Beyotime). Incubate the suspension on ice for 30 min, and then the cell lysate was centrifuged at low temperatures according to the instructions to separate the protein fraction. The Omni-Easy Instant BCA Protein Assay Kit (Epizyme, China) was used to measure the protein concentration in the supernatants. Following the proteins' mixture with loading buffer and 10-min heating to 100°C, they were examined using sodium dodecyl sulfate-polyacrylamide gel electrophoresis (SDS-PAGE)(Epizyme). Then, different quantities of 20 μg of each sample were loaded into the designated wells of SDS-PAGE gels with varying concentrations (12.5%, 10%, and 6%) for separation and subsequent analysis. Following their separation, the proteins were placed onto polyvinylidene fluoride (PVDF) membranes (Millipore) that had been previously moistened. The process of blocking was employed to halt non-specific binding. The membranes were subjected to this by applying a 5% bovine serum albumin (BSA) solution (Pumoke, China). With that, the blocking solution was subsequently permitted to incubate on the membranes at room temperature for 2 h. They were exposed to antibodies that selectively bind to the following targets: mTOR (HuaBio, C hina), p-MTOR (HuaBio, China), AKT (ABclonal), p-AKT (ABclonal, China), β-actin (ABclonal), VEGFA (ABclonal), TSG101 (Noninbio, China), Calnexin (Abcam, USA), and CD63 (Noninbio). The primary antibodies for p-AKT and mTOR were diluted at a 1:500 ratio, while a 1:1000 dilution of the remaining primary antibodies was applied. The incubation is conducted at 4°C for a duration of 12 h. Once the PVDF membranes were cleaned with TBST buffer, the relevant HRP-labeled Goat Anti-Rabbit IgG(H+L) (Beyotime) or HRP-labeled Goat Anti-Mouse IgG(H+L)(Beyotime) was diluted at a 1:10000 ratio. The membranes were incubated together for 1 h at room temperature to improve the recognition of protein bands. The Omni-ECL Enhanced Pico Light Chemiluminescence Kit (Epizyme) was employed for observing the protein bands, and the Bio-Rad Chemidoc system (Bio-Rad, USA) was employed for data recording.

### Cell Proliferation Assay

Twenty thousand HUVECs were resuspended, implanted into a 6-well plate, and then cultured in a cell culture incubator for a duration of 12 h to facilitate cell attachment. The cells were subjected to PSC-exo at doses of 10 μg/ml, 30 μg/ml, and 50 μg/ml after a 24 h hunger. After co-cultivating with PSC-exo for both 24 h and 48 h, following the removal of the original medium, PBS wash was utilized for washing the cells. Upon the completion of the treatment, each well was given 550 μl of CCK-8 solution and incubated for 30 min. Five wells in the 96-well plate were filled with 100 μl of sample from each well. Absorbance at 450 nm was determined using an enzyme marker.

### Transwell Migration Assay

Before the commencement of the assay, the HUVECs were subjected to a 24 h starvation period. At the outset of the experiment, the cells were resuspended using a serum-free medium and counted to obtain 40,000 cells. Subsequently, the cells were added to 200 μl of serum-free medium and mixed with an equal amount of PBS or 50 μg/ml of PSC-exo. Concurrently, 500 μl of medium containing 20% FBS was combined with an equal volume of PBS or 50 μg/ml of PSC-exo, and the resulting mixture was added to a 24-well plate. Subsequently, the cells were added to the upper chamber, after which the transwells were placed into wells containing 500 mL of medium. The 24-well plate was then incubated in a cell culture incubator for 24 h. At the designated time point, the transwells were removed and fixed in a 4% paraformaldehyde (PFA) solution (Beyotime) for 30 min. Thereafter, cells in the bottom layer were stained with crystal violet (Beyotime) for 10 min. The stained cells were quantified using an inverted confocal microscope (Olympus, Japan) and ImageJ software (NIH, USA).

### Transwell Invasion Assay

A solution with a ratio of 1:8 was prepared by adding 60 μl of Matrigel (BD Biocoat, USA), which was then applied to the transwell inserts. The well plate with transwell inserts was incubated in a cell incubator for 3 h to allow the Matrixgel to separate into a film. Medium containing 20% FBS was given into the well plate wells in an amount of 500 μl, and 200 μl of medium without serum was applied to seed 50000 HUVECs in the upper chamber. The control group received PBS in both chambers, while the experimental group received 50 μg/ml of PSC-exo in both chambers. The cells in the upper chamber were gently withdrawn following a 24-h incubation period. Following fixing for 30 min with 4% PFA (Beyotime), for 10 min, the cells that could be seen through the bottom were stained with crystal violet (Beyotime). The stained cells were quantified using an inverted confocal microscope (Olympus, Japan) and ImageJ software (NIH).

### Tube Formation Assay on HUVECs In Vitro

Thirty min were spent at 37°C incubating 60 μl of uniformly dispersed Matrigel (BD Biocoat) in a 96-well plate. 60,000 HUVECs went into cultivation at an amount of 100 μl of medium after being starved for 12 h. PSC-exo were applied to the cells in the group experimenting with a 50 μg/ml dose, whereas the PBS comparable volume was given to the control group. Tube formation was apparent by means of an inverted microscope (Olympus) after 3 h of incubation, and photographs were captured. Quantification of tube number and branching length was performed using the ImageJ (NIH) Angiogenesis Analyzer plugin, enabling objective analysis of angiogenic characteristics.

### In-Vivo Matrigel Plug Angiogenesis Assay

BALB/c nude mice (five weeks old) were subcutaneously injected with 400 μl of Matrigel containing 300,000 HUVECs cells, along with either PSC-exo (*n* = 4) or PBS (*n* = 4). After 10 days, the Matrigel plugs were collected and processed. The plugs were fixed, embedded in paraffin, and sectioned. Angiogenesis was assessed using H&E staining for morphological analysis and CD31 immunostaining to identify ECs. ImageJ (NIH) software was utilized to analyze the angiogenic features quantitatively.

### Ethics Statement

The Institutional Animal Care and Use Committee (IACUC) of Chongqing Medical University approved the animal operations used in this study (Approval Number: IACUC-CQMU-2023-0239). Throughout the research project, ethical standards and animal welfare have been a priority.

### Statistical Analysis

Statistical analysis was performed using GraphPad Prism 9 (GraphPad, USA). The data presented are the mean± SEM of a minimum of three independent samples. A two-way ANOVA was utilized for comparisons between groups, whereas the differences between the two groups were determined by the *t*-test. To establish statistical significance, an important difference between the groups under comparison was indicated by a significance level of 0.05.

## Results

### Exosomes Characterization from PSCs

**Fig. 1:**
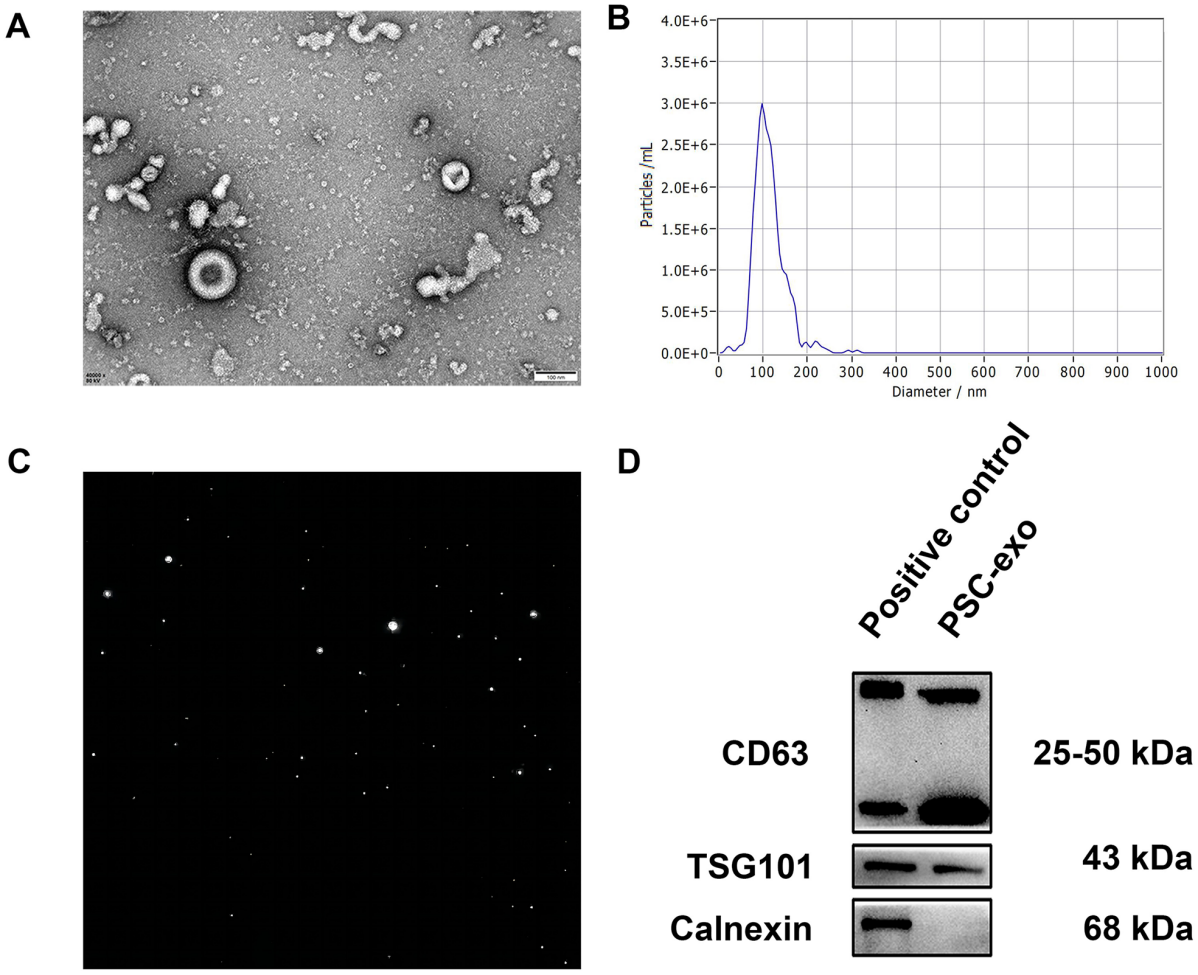
Isolation and identification of exosomes from PSCs. (**A**) The TEM images reveal the typical cup-shaped morphology of exosomes. (**B**) Analysis of exosome size distribution using NTA. (**C**) The image of the filtered exosomes. (**D**) Western blotting examination of exosome surface markers CD63 and TSG101, with Calnexin as a negative control. Scale bar: 100 μm.

The exosomes were isolated and analyzed using TEM, NTA, and Western blotting. The TEM pictures displayed the distinctive cup-shaped morphology of the particles. The NTA results indicated that the particle diameters are concentrated around at 100 nm. The Western blotting results indicated the presence of both CD63 and TSG101 proteins, although the Calnexin protein was not (Fig. 1). All the above results demonstrated that the exosomes were successfully extracted.

### In Vitro, Exosomes Enhance the Proliferation, Migration, and Invasion of HUVECs

**Fig. 2:**
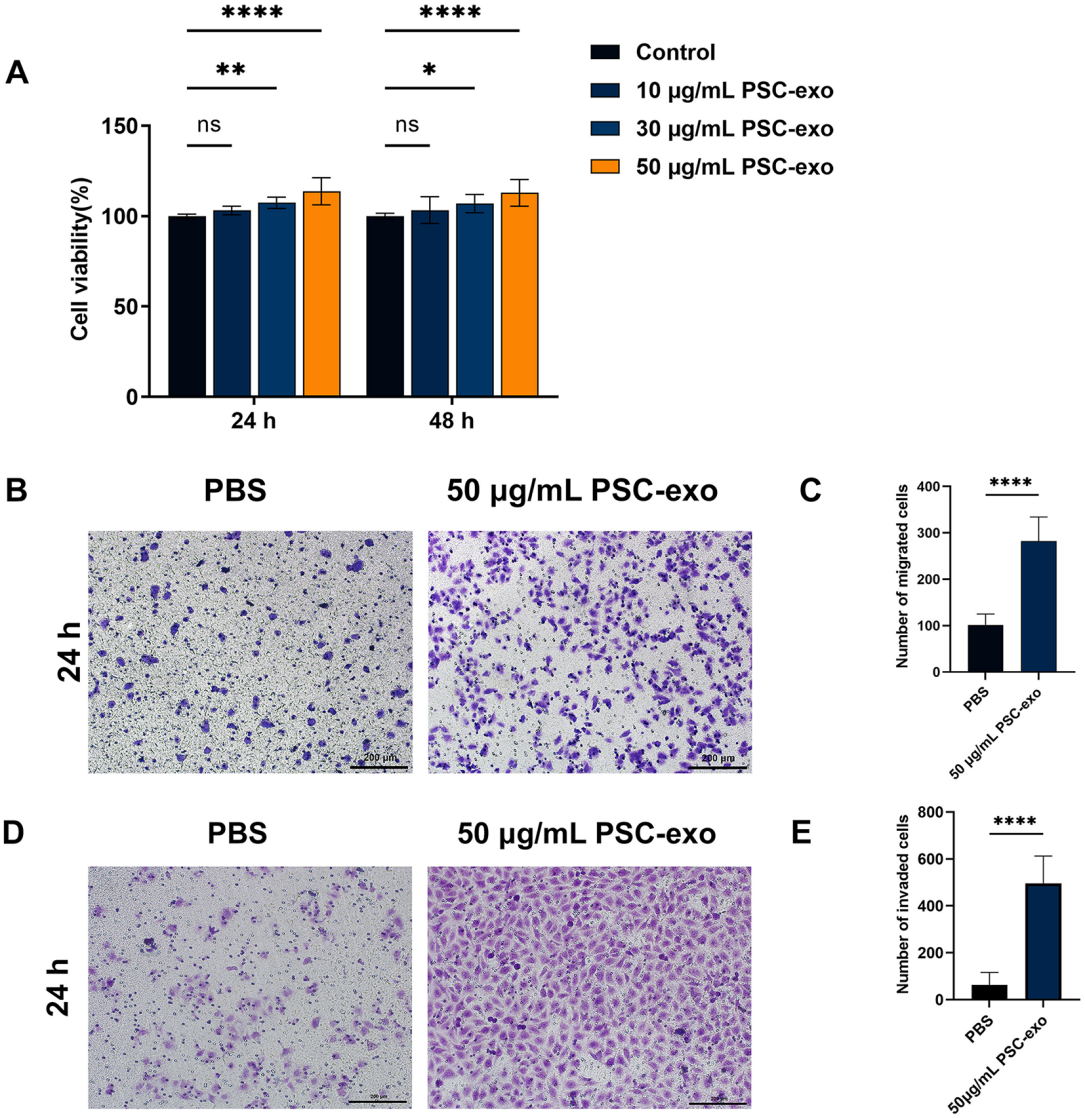
PSC-exo affect the proliferation, migration, and invasion of HUVECs. (**A**) At 24 and 48 h, the relative cell viability of HUVECs co-cultured with PSC-exo at 10, 30, and 50 μg/ml doses. (**B, C**) Relative cell migration of HUVECs cocultured with either PBS or PSC-exo at doses of 50 μg/ml at 24 h. (**D, E**) Relative cell invasion of HUVECs co-cultured with either PBS or PSC-exo at a 50 μg/ml dose at 24 h. The data represents the mean ± SEM from a minimum of three independent replicates. ns, not significant; *p < 0.05, **p < 0.01, ****p < 0.0001. Scale bar:200 μm.

CCK-8 assay's findings demonstrated a steady rise in cell viability as PSC-exo concentrations increased. At 24 h and 48 h after adding PSC-exo, there was no statistically significant change in cell proliferation between the control group and those treated with a concentration of 10 μg/ml PSC-exo, according to the results of the CCK-8 assay. Nonetheless, a noteworthy distinction was noted at a concentration of 30 μg/ml. The difference was even more pronounced at 50 μg/ml doses (Fig. 2A). A comparison of the control group with the PSC-exo group revealed a significant increase in the migration ability and the number of migrating cells of the latter after 24 h.(Fig. 2B and 2C). In addition, the invasiveness of HUVECs cultured with PSC-exo at a 50 μg/ml dose was considerably higher than in the control group (Fig. 2D and 2E).

### In Vitro, Exosomes Promote Tube Formations of HUVECs

**Fig. 3:**
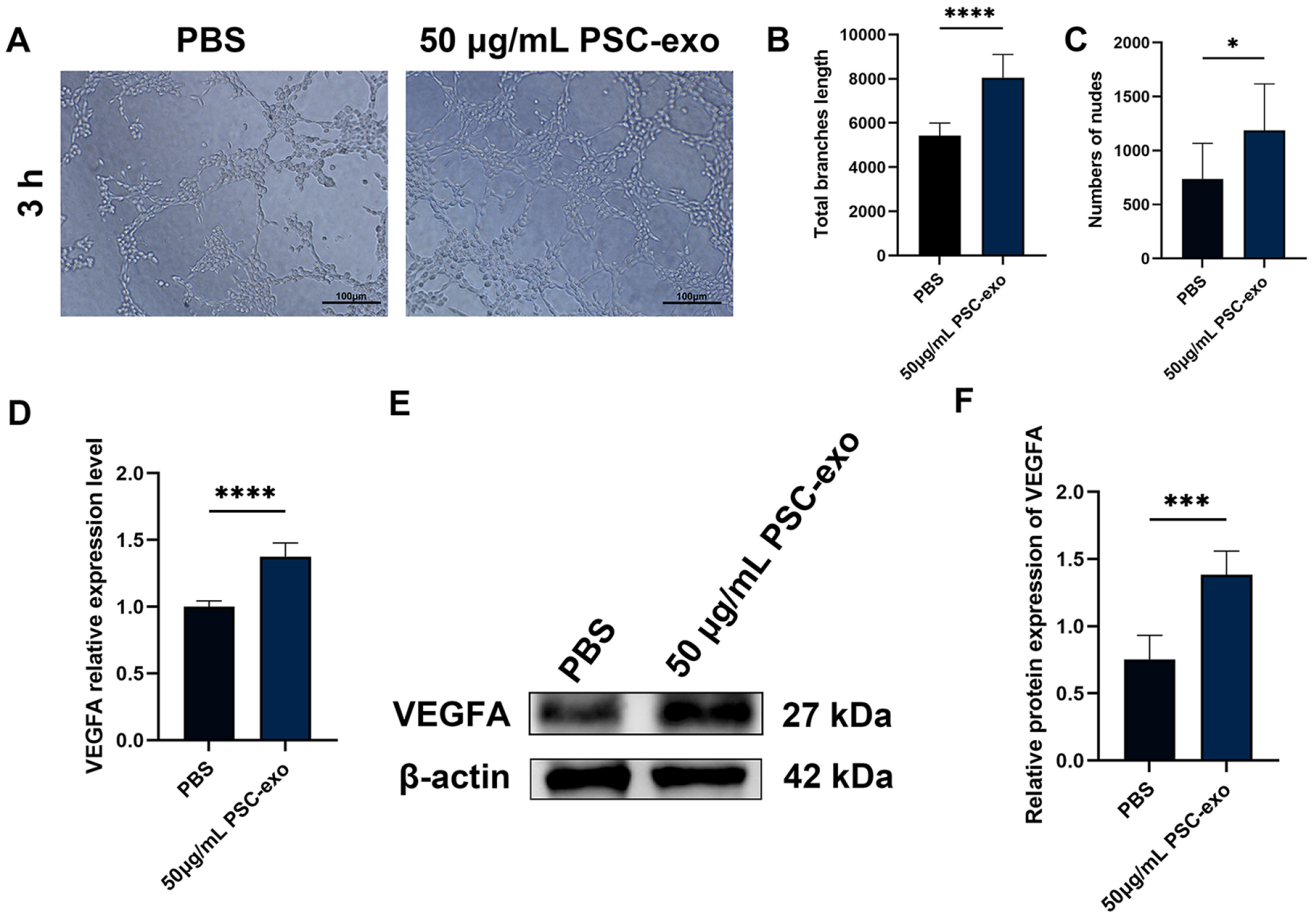
Effects of PSC-exo on tube formation and VEGFA expression. (**A-C**) The numbers of nodes and total branches length of tube formation in HUVECs co-cultured with either PBS or PSC-exo at 50 μg/ml doses after 3 h. (**D**) VEGFA expression levels in HUVECs co-cultured with PBS or 50 μg/ml PSC-exo for 48 h, as detected by qRT-PCR. (**E, F**) VEGFA protein levels in HUVECs co-cultured with PBS or 50 μg/ml PSC-exo for 48 h, as detected by Western blotting. The data represents the mean ± SEM from a minimum of three independent replicates. *p < 0.05, ***p<0.001, ****p<0.0001. Scale bar: 100 μm.

A tube formation assay was conducted on HUVECs, and the findings indicated that the addition of 50 μg/ml PSC-exo significantly enhanced the cells' ability to form tubes. When comparing the PSC-exo group to the control group, it was observed that the former had more tubes and longer branches. (Fig. 3A-3C).

### The Expression of VEGFA Is Upregulated by PSC-Exo

The addition of PSC-exo at a dose of 50 μg/ml significantly increased VEGFA expression after 48 h co-cultured with HUVECs, as validated by qRT-PCR and Western blotting studies (Fig. 3D-3F).

### PSC-Exo Stimulate the AKT/mTOR Signaling Pathway, which in turn Promotes Angiogenesis

**Fig. 4:**
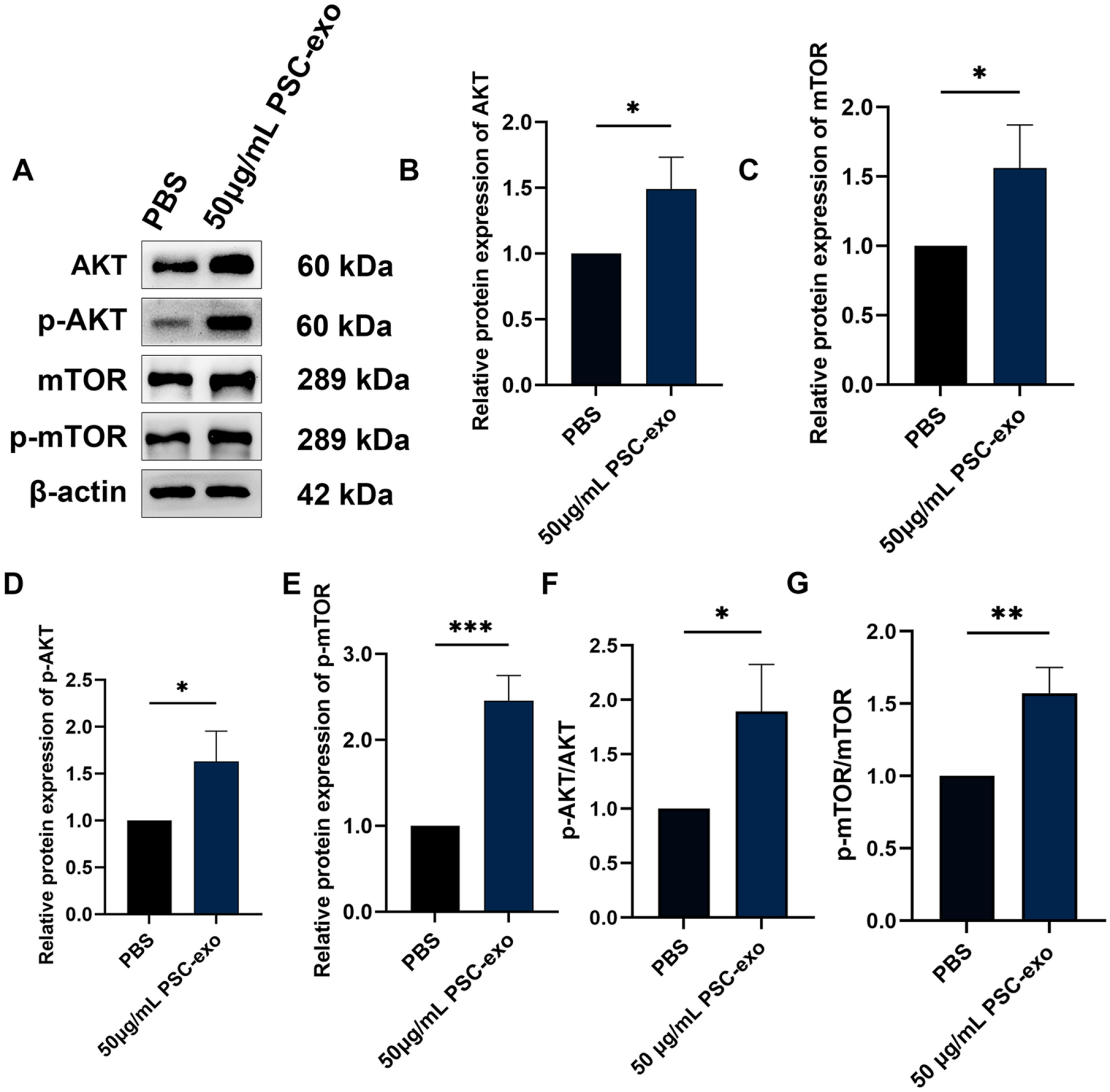
Effects of PSC-exo on the AKT/mTOR signaling pathway. (**A-E**) Western blotting assay showing AKT, p-AKT, mTOR, and p-mTOR protein levels in HUVECs co-cultured with PBS or 50 μg/ml PSC-exo. (**F**) AKT activation level. (**G**) mTOR activation level. The data represents the mean ± SEM from a minimum of three independent replicates. *p < 0.05, **p < 0.01, ***p<0.001.

To explore the mechanisms by which PSC-exo promotes proliferation, migration, invasion, and angiogenesis in HUVECs, we studied the expression and phosphorylation of critical proteins in the AKT/mTOR signaling pathway. The Western blotting analysis showed that PSC-exo markedly increased the expression levels of AKT, p-AKT, mTOR, and p-mTOR (Fig. 4A-4E). Additionally, the PSC-exo group displayed significantly higher p-AKT/AKT and p-mTOR/mTOR ratios compared to the control group, indicating a pronounced activation of the AKT and mTOR pathways in the PSC-exo group. These findings suggest that the PSC-exo group displayed a heightened proficiency in stimulating AKT and mTOR signaling, two critical pathways involved in a multitude of cellular functions and signaling cascades (Fig. 4F and 4G).

### PSC-Exo Promote Angiogenesis In Vivo

**Fig. 5:**
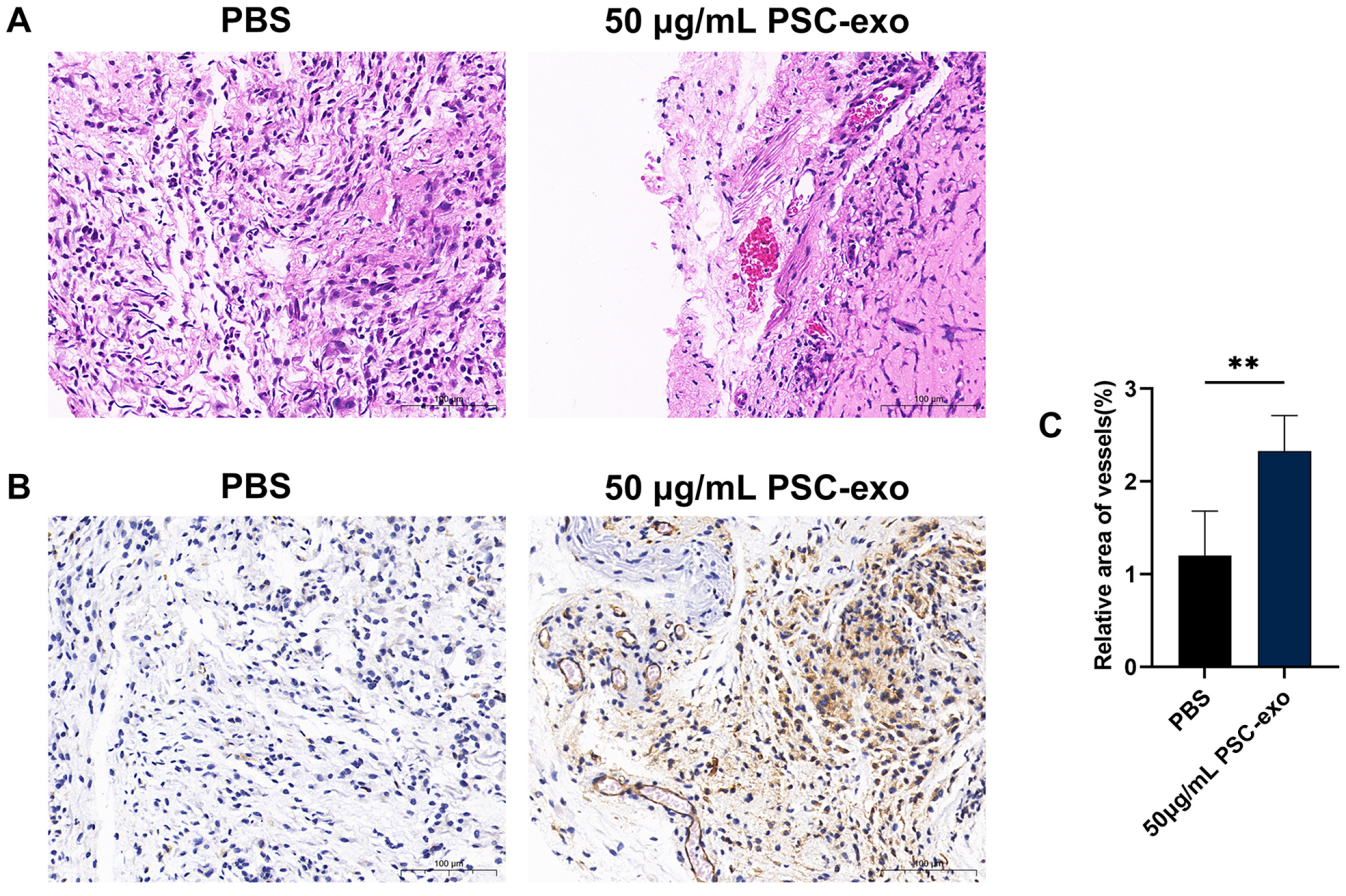
The effect of PSC-exo on angiogenesis in Matrigel plugs. (**A**) HE staining of the Matrigel plugs. (**B, C**) CD31 positive areas in Matrigel plugs and quantitative results. Data are reported as mean ± SEM, and all experiments were conducted with a minimum of three replicates. **p < 0.01. Scale bar: 100 μm.

To assess the pro-angiogenic potential of exosomes in an in vivo setting, we performed Matrigel plug experiments in BALB/c nude mice, comparing groups with and without exosomes. The evaluation of the results involved histological analysis using H&E staining and CD31 immunohistochemistry. The analysis demonstrated a notable increase in the number of vessels or capillary-like structures in the presence of exosomes compared to the control group treated with PBS (Fig. 5).

## Discussion

*E. multilocularis* invades neighboring tissues and organs in a manner similar to malignant tumors, and can also spread and metastasize through blood vessels. The growth of *E. multilocularis* is facilitated by the development of new blood vessels (neovascularization) [22]. Exosomes from different tumor origins can activate ECs and increase vascular permeability, promoting tumor metastasis and dissemination[23-26]. However, the function and mechanism of *E. multilocularis* exosomes in regulating angiogenesis remain to be elucidated. This study demonstrated that PSC-exo can stimulate angiogenesis in HUVECs. The investigation revealed that PSC-exo activated the AKT/mTOR signal transduction pathway by regulating the expression of VEGFA, thereby promoting the proliferation, migration, invasion, and tube-forming ability of HUVECs in vitro. These findings indicate that PSC-exo play a pivotal role in the formation of the vascular network.

In addition, previous studies have demonstrated a significant association between VEGFA and overexpression and angiogenesis in *E. multilocularis* [27, 28]. VEGFA, which is part of the vascular endothelial growth factor family, is secreted by tumor cells and plays a crucial factor in promoting neo-angiogenesis [29]. Therefore, VEGFA plays a crucial role in neointima formation. The results demonstrated a notable elevation in VEGFA levels following the co-culture of PSC-exo with HUVECs, in alignment with previous studies. The observed increase may be attributed to the regulatory role of bioactive molecules released by PSC-exo, including proteins [30], mRNAs [31], miRNAs [32], and long non-coding RNAs (lncRNAs) [33]. Moreover, VEGFA binds to VEGFR2 receptors on vascular ECs, leading to receptor dimerization, activation of protein kinases, and subsequent autophosphorylation, driving the basic processes of angiogenesis (migration, proliferation, tube formation) [34]. Consequently, the observed increase in VEGFA expression may contribute to the enhanced angiogenic capacity exhibited by HUVECs in response to PSC-exo stimulation.

Further investigation was undertaken to gain a deeper understanding of the downstream pathways affected by VEGFA. It has been demonstrated that VEGFA binding to its receptor can activate some downstream signaling pathways, including the AKT/mTOR pathway [29], the SRC pathway [35], the ERK/MAPK pathway [36], and others. Prior research has suggested that the AKT/mTOR signaling pathway is crucial in fundamental biological mechanisms implicated in the growth and progression of tumors, including cell proliferation [37], programmed cell death [38], and angiogenesis [39]. AKT, acting as a central signaling molecule, intricately regulates angiogenesis by activating mTOR through phosphorylation or direct activation. This activation of mTOR facilitates protein translation, and synthesis, as well as cell growth and metabolism, thereby contributing to the overall process of angiogenesis [40]. Additionally, mTOR regulates VEGFA production at transcriptional and translational levels [41], creating a positive feedback loop. It has previously been demonstrated that VEGFA stimulates the formation of new blood vessels by activating the AKT/mTOR signaling pathway downstream [42]. The findings of this study indicated that PSC-exo stimulation modulates the phosphorylation levels of AKT and mTOR, thereby promoting angiogenesis in HUVECs. Therefore, the stimulation of the AKT/mTOR pathway by PSC-exo is shown to encourage angiogenesis in *E. multilocularis*, which in this case facilitates the development of blood vessels.

The identification of VEGFA as a principal tumor angiogenic factor has resulted in the development of numerous drugs that target it or its receptors, such as VEGF blockers [43, 44]. This study has revealed that PSC-exo has the potential to regulate VEGFA and its downstream signaling pathways. Based on our preliminary studies on the function of PSC-exo, we propose a new therapeutic scenario for intervening in the angiogenic process of AE by regulating the release or function of PSC-exo to indirectly modulate VEGFA and its downstream signaling pathways.

Ultimately, this research affirms that PSC-exo have a role in enhancing neovascularization in *E. multilocularis* infection. PSC-exo at a dose of 50 μg/ml were found to stimulate angiogenesis-related functions in HUVECs by modulating VEGFA and its downstream AKT/mTOR signaling pathways. This has subsequently been validated through in vivo experimentation. Although this study has not definitively identified the exact molecule responsible for the observed effects of PSC-exo, it provides valuable insights into the molecular mechanisms of PSC-exo in angiogenesis. Future studies should aim to identify a more comprehensive control group to further explore the specific role of PSC-exo. This study is beneficial in improving our understanding of neovascularisation in AE. While further studies are necessary to confirm these potential therapeutic targets and to explore the feasibility of their clinical application, this study provides useful clues and directions for future treatment of AE.
